# Influence of language and ancestry on genetic structure of contiguous populations: A microsatellite based study on populations of Orissa

**DOI:** 10.1186/1471-2156-6-4

**Published:** 2005-02-05

**Authors:** Sanghamitra Sahoo, VK Kashyap

**Affiliations:** 1 National DNA Analysis Centre, Central Forensic Science Laboratory, 30, Gorachand Road, Kolkata-700 014 India

## Abstract

**Background:**

We have examined genetic diversity at fifteen autosomal microsatellite loci in seven predominant populations of Orissa to decipher whether populations inhabiting the same geographic region can be differentiated on the basis of language or ancestry. The studied populations have diverse historical accounts of their origin, belong to two major ethnic groups and different linguistic families. Caucasoid caste populations are speakers of Indo-European language and comprise Brahmins, Khandayat, Karan and Gope, while the three Australoid tribal populations include two Austric speakers: Juang and Saora and a Dravidian speaking population, Paroja. These divergent groups provide a varied substratum for understanding variation of genetic patterns in a geographical area resulting from differential admixture between migrants groups and aboriginals, and the influence of this admixture on population stratification.

**Results:**

The allele distribution pattern showed uniformity in the studied groups with approximately 81% genetic variability within populations. The coefficient of gene differentiation was found to be significantly higher in tribes (0.014) than caste groups (0.004). Genetic variance between the groups was 0.34% in both ethnic and linguistic clusters and statistically significant only in the ethnic apportionment. Although the populations were genetically close (F_ST _= 0.010), the contemporary caste and tribal groups formed distinct clusters in both Principal-Component plot and Neighbor-Joining tree. In the phylogenetic tree, the Orissa Brahmins showed close affinity to populations of North India, while Khandayat and Gope clustered with the tribal groups, suggesting a possibility of their origin from indigenous people.

**Conclusions:**

The extent of genetic differentiation in the contemporary caste and tribal groups of Orissa is highly significant and constitutes two distinct genetic clusters. Based on our observations, we suggest that since genetic distances and coefficient of gene differentiation were fairly small, the studied populations are indeed genetically similar and that the genetic structure of populations in a geographical region is primarily influenced by their ancestry and not by socio-cultural hierarchy or language. The scenario of genetic structure, however, might be different for other regions of the subcontinent where populations have more similar ethnic and linguistic backgrounds and there might be variations in the patterns of genomic and socio-cultural affinities in different geographical regions.

## Background

Human society in a geographic area develops when colonizing populations bring along with them different languages, cultures and technological advancements over a period of time. As more populations migrate to settle in the same area, they are either eliminated, subjugated or absorbed [1]. In India, majority of incoming populations have been absorbed, forming heterogeneous and complex human societies. A few have subjugated the subservient cultures to establish a hierarchical caste system or have totally isolated some groups such as tribes, which still remain outside the social boundaries. This practice has enriched India with populations having varied socio-cultural and linguistic diversities that have flourished independently, nurtured by the vast geographical and ecological regime [2]. Studies based on various DNA markers on diverse populations occupying different geographical areas of the Indian subcontinent have revealed much about the presence of large extent of human genetic variation [3-10] and the distinct genetic difference between castes and tribal populations of India [11-13]. These studies, however fail to characterize the structure of populations in geographic contiguity, where populations with different language and social hierarchies cohabit together. Although distinct social demarcation between castes and tribes is well established, the origin of a few populations of India still remains controversial. Though many castes are known to have tribal origins [14], nevertheless their assessment with polymorphic DNA markers still remains incomplete.

This study aims to understand the genetic diversity of populations of Orissa and examines the role of language and genetic origin on structure of populations inhabiting the same geographic region and evaluates some of the suggested population histories from a molecular perspective. Orissa is a coastal state in the southeast region of India, which is occupied by population groups having varied ethnicity, belonging to different strata of the hierarchical caste system and speaking languages belonging to different linguistic families. Its strategic geographic location between Northern plains and peninsular Southern India and cultures assimilated during the 4^th ^– 5^th ^century B.C. from southeast Asian countries of Java, Sumatra, Brunei and Indonesia [15] have enriched the socio-cultural diversity of contemporary populations of Orissa. The extant populations of the region can be broadly classified into two major social groups; castes and tribes. Brahmin, Khandayat, Karan and Gope comprise a large section of Indo-European speaking caste populations of Orissa, whose position in hierarchical caste system is governed by occupation and where ancestry is patrilineal. Brahmins form the priestly class who occupy uppermost strata in the caste hierarchy, with historical accounts that trace their migration from upper Gangetic regions of north India. Next in hierarchy is the Kshatriyā – a warrior group comprising the Khandayats; followed by Karans (Kayasthā), record keepers and Gope are cattle-breeders who occupy the subsequent strata in caste system [16]. Other than caste groups, tribes constitute a large number of aboriginal Australoid populations of Orissa who are predominantly forest dwellers, most of them having their own dialects. Linguistically, the tribal groups of the region can be categorized into three of the four major language families spoken in India: Indo-European, Austro-asiatic and Dravidian. Kharia, Juang, Gadaba, Ho, Munda and Saora are among few of the most ancient tribes whose dialects belong to the Austro-asiatic linguistic family, while those of Paroja, Oraon and Kondh belong to the Dravidian linguistic group [16]. Of these populations, only a few (Paroja, Agharia, Gaud, Tanti) have been included in studies using DNA markers to get a perspective of the overall genetic diversity present in the country [8,11,12]. Hence, to understand the genetic constitution of these ethnically and linguistically diverse populations, we have used autosomal microsatellites, genetic markers with proven precision in deciphering genomic diversity and affinities of human populations [17].

Microsatellites or short tandem repeats (STRs) are most extensively used for elucidating the genetic diversity and evolution of human populations because of their abundance and prevalence in the genome, high level of polymorphism and amenability to automation [18-23]. High mutation rates of STR loci facilitate inferences to be drawn about population substructure and short-term evolutions and to make a more reliable and precise estimation of phylogenetic relationships among populations both at racial and continental levels [24-29]. Also, most questions of anthropological interest involve processes occurring over relatively short time periods, during which substantial genetic drift and migration may occur but fewer mutations get accumulated. These minor changes are easily detected using STR markers rather than bi-allelic markers, where mutations accumulate slowly through evolutionary time. STR markers are therefore markers of choice for this study, which involves closely related populations that share similar ethnicity, language, culture or history of origin.

**Figure 1:**
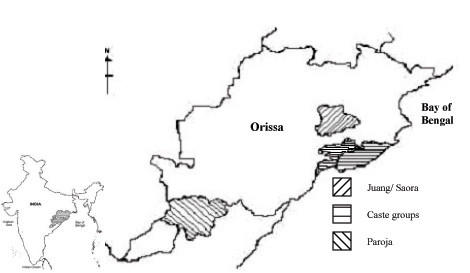
Geographical map of Orissa showing the location of sample collection

In this study, we have examined variation at 15 autosomal STR loci in a sample of 404 individuals from Orissa (Table 1, Figure 1) and compared the results with previously published data from other regions of the Indian subcontinent. Our aim was (i) to assess the genetic diversity and relationship of populations of Orissa with other Indian populations, and (ii) to find out the role of language and ancestry, if any, on genetic structure of populations living in geographic contiguity. This study also allows a finer resolution of population history of the region than has hitherto been possible.

**Table 1:** Demographic characterization of the seven studied populations of Orissa

	**POPULATION**	**CODE**	**n**	**SOCIAL HIERARCHY**	**ETHNICITY**	**LANGUAGE GROUP**
1	ORIYABRAHMIN	OB	57	Upper Caste	Indo-Caucasoid	Indo-European
2	KARAN	KR	62	Middle Caste	Indo-Caucasoid	Indo-European
3	KHANDAYAT	KY	62	Middle Caste	Indo-Caucasoid	Indo-European
4	GOPE	GP	60	Lower Caste	Indo-Caucasoid	Indo-European
5	JUANG	JU	50	Tribe	Proto-Australoid	Austro-Asiatic
6	SAORA	SA	35	Tribe	Proto-Australoid	Austro-Asiatic
7	PAROJA	PR	78	Tribe	Proto-Australoid	Dravidian

## Results

### Nature and extent of allelic diversity

The distribution of allele frequencies and tests of Hardy-Weinberg Equilibrium (HWE) on the seven populations of Orissa have been previously reported [30,31]. Except for Saora, all other studied populations were found to be in HWE. Saora showed significant departures from HWE at three analysed parameters (p < 0.05 for exact test and homozygosity test; p < 0.1 for log-likelihood ratio test) and a lower heterozygosity value (0.571) compared to the expected estimates of allele frequencies at D3S1358 locus. Number of alleles and most common alleles at the fifteen STR loci along with gene diversity of each of the studied seven populations are shown in Table 2a, 2b and 3. The most common alleles at each of the 15 STR loci were shared between 2–4 populations. These results agree with the analysis of 4 STR loci (CSF1Po, TPOX, THO1 and vWA) reported by Mukerjee et al, 1999 on three populations of Orissa (Agharia, Gaud and Tanti). The number of alleles observed in the studied population and heterozygosity values (0.615–0.967) indicate that the selected STR markers are highly polymorphic in all populations and that genetic variability within populations is significantly high across populations with mean gene diversity of 81%.

**Table 2a:** Allelic Diversity at 8 of 15 STR loci describing the extent of variation within the populations of Orissa

	**D3S1358**		**THO1**		**D21S11**		**D18S51**		**D5S818**		**D13S317**		**D7S820**		**D16S539**	
	
	Alleles	MCA	Alleles	MCA	Alleles	MCA	Alleles	MCA	Alleles	MCA	Alleles	MCA	Alleles	MCA	Alleles	MCA
Oriya Brahmin	6	15	6	6	11	29	11	15	6	12	8	11	7	11	6	11
Khandayat	6	15	6	9	11	29	10	14	7	11	8	12	8	11	8	11
Karan	7	15	5	6	10	32	11	14	6	11,12	8	12	7	11	8	11
Gope	8	15	6	9	10	32	12	14	8	12	8	11	8	11	7	11
Juang	6	15	6	9	8	29	10	15	6	11	7	11	7	8	7	11
Paroja	6	15	6	9	9	32	11	15	6	11	8	8	6	11	7	12
Saora	6	16,17	6	9	8	30	11	9	6	11	6	8	7	11	6	12

* Alleles = No. of Alleles, MCA = Most Common Allele

**Table 2b:** Allelic Diversity at 7 of 15 STR loci describing the extent of variation within the populations of Orissa

	**CSF1Po**		**vWA**		**D8S1179**		**TPOX**		**FGA**		**Penta E**		**Penta D**	
	
	Alleles	MCA	Alleles	MCA	Alleles	MCA	Alleles	MCA	Alleles	MCA	Alleles	MCA	Alleles	MCA
Oriya Brahmin	7	12	7	17	11	14	6	8	13	24	14	11	6	11
Khandayat	7	12	8	17	9	10,15	7	11	16	24	14	12	8	11
Karan	7	12	7	16	9	10	5	11	13	22	15	11	9	11
Gope	8	12	7	17	8	14	5	11	11	24	17	11,12	9	9
Juang	6	12	6	17,18	8	15	5	11	11	19,22	13	12	8	10
Paroja	5	12	8	14	8	10	5	11	14	23	18	11	9	10
Saora	5	11	5	16	8	13	5	8	10	21	15	11	8	9

* Alleles = No. of Alleles, MCA = Most Common Allele

**Table 3:** Gene diversity estimated from 15 autosomal STR loci describing the total variation within the seven studied populations of Orissa

**Population**	**Gene Diversity**
Oriya Brahmin	0.816 ± 0.017
Khandayat	0.811 ± 0.015
Karan	0.817 ± 0.018
Gope	0.815 ± 0.014
Juang	0.804 ± 0.015
Paroja	0.811 ± 0.013
Saora	0.810 ± 0.016

### Extent of differentiation between populations

To quantify the amount of genetic diversity that exists among populations, F_ST _was calculated separately for caste groups and tribes. The coefficient of gene differentiation was found to be significantly higher in tribes (0.014) than caste groups (0.004). Combining all seven populations yielded an F_ST _of 1%, demonstrating low level of population differentiation within Orissa. All values of F_ST _were significantly different from zero (p < 0.05). Analysis of molecular variance (AMOVA) presented in Table 4, revealed that as a single group, a large extent of genetic variation (98.98%) was present within the populations of the region. To determine how the residual genetic variance was compartmentalized, we grouped the populations into (i) caste and tribes, (ii) linguistic groups; Indo-European speaking caste populations (Oriya Brahmins, Karan, Khandayat, Gope), Austro-Asiatic speakers Juang and Saora and Dravidian speaking, Paroja and (iii) according to their origins as suggested by historical accounts. The genetic variance between the groups varied from 0.25% to 0.34% and was equally distributed in both ethnic and linguistic clusters, but statistically significant only in the ethnic apportionment.

**Table 4:** Variance in populations of Orissa due to ethnicity, language and history of origin at three different levels of hierarchy analysed with 15 autosomal STR loci

**Basis**	**Grouping**	**Populations in group**	**% of total variance (p values)**		
			Within population	Between population within group	Between groups

Ethnicity	Castes vs Tribes	(OB, KY, KR, GP) vs (JU, PR, SA)	98.84 (0.00)	0.83 (0.00)	0.34 (0.03)
Language	Indo-European vs Austro-Asiatic vs Dravidian	(OB, KY, KR, GP) vs (JU, SA) vs (PR)	98.86 (0.00)	0.79 (0.00)	0.34 (0.05)
History of Origin	European vs Austro-Asiatic vs Admixed Gene Pool	(OB, KR) vs (JU, SA) vs (KY, GP, PR)	98.91 (0.00)	0.84 (0.00)	0.25 (0.06)

**Figure 2:**
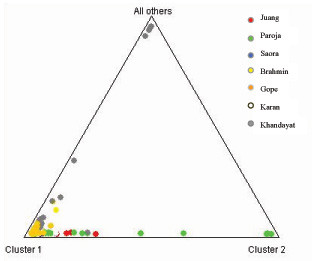
Assignment of samples from seven populations of Orissa to genetic clusters inferred from the STRUCTURE analysis for K = 3.

Because the amount of genetic variance between groups was found to be low, we also used clustering algorithm implemented in STRUCTURE analysis (Figure 2) to explore the population structure and relationship among these geographically contiguous but socially and linguistically disparate populations. When the populations were analysed assuming no admixture model and K varying from 1 to 7, only a single distinct genetic cluster could be found with the highest log likelihood value at K = 3. Most of the individuals of the seven populations clustered in cluster 1 and did not split into distinct clusters corresponding to their population affinities. A few of the individuals of Paroja and Khandayat were found in cluster 2 and 3 respectively.

### Genetic relationship among populations

**Figure 3:**
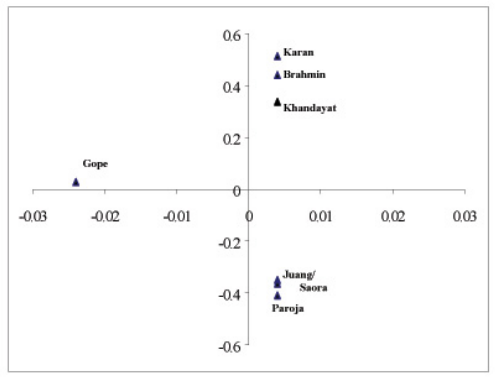
PC plot for the seven populations of Orissa from centroid based on fifteen microsatellite loci

The inter-population genetic relationship among Brahmins, Khandayat, Karan, Gope, Juang, Saora and Paroja was determined using principal component analysis. The plot (Figure 3) of principal component (PC) depicts population configurations in accordance with their ethnic affiliations. Together, the first two principal coordinates described almost 99.9% of the variance in the distance matrix. The caste populations (Brahmins, Khandayat and Karan) and the three tribal populations of Juang, Saora and Paroja were distinctly separated by the first component of the distance matrix. All the caste populations were found to cluster in the upper right quadrant while the tribes distinctly occupied the lower right quadrant. The only discordance was position of Gope, where this population was genetically separate from the other studied caste populations in the PC plot.

**Figure 4:**
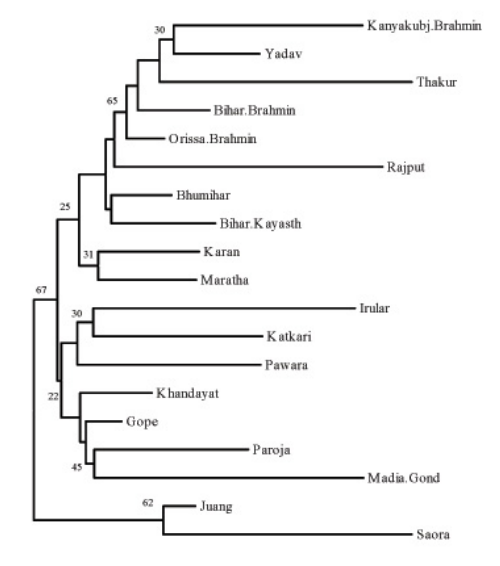
Neighbor-joining tree of genetic distances (D_A_) based on fifteen microsatellite loci among studied populations of India

The Neighbour-Joining (NJ) tree (Figure 4) gives a graphical representation of genetic distance of Orissa populations from populations of Bihar [32,33], Uttar Pradesh [34], Maharastra [35,36] and Tamil Nadu [37], belonging to similar rank and occupational affiliation in the caste hierarchy. The genetic closeness exhibited by Brahmins of Orissa to those of North India (NJ tree; Figure 4) was clearly discernible, supported by moderately high bootstrap values. While Karan belonging to the next level of hierarchy in the caste system showed similarity to Maratha, a warrior group of Maharastra; Khandayats and Gope depicted affinity to the tribal populations (Figure 4). Paroja, a Dravidian linguistic group, demonstrated affinity with Gonds, and the two Austro-Asiatic speakers Juang and Saora distinctly branched out in the phylogenetic tree.

## Discussion

India is a remarkable representation of a large segmented society that harbours rich genetic diversity within its human populations and offers myriads of attributes to study the various factors influencing demographics of human populations. It is of particular interest to study patterns of genetic affinities among endogamous groups inhabiting small geographical regions within the subcontinent because of their diverse origins and interethnic admixtures.

We have typed a set of fifteen polymorphic autosomal microsatellite markers in linguistically and socially divergent populations with different histories of origin to elucidate the genetic diversities and affinities among them and to understand the role of genetic origin and language on the genetic structure of populations living in geographic contiguity. The most distinctive feature of our study was the clear delineation between castes and tribes, as was evident from both multivariate and phylogenetic analyses (Figure 3 and 4 respectively). The tribes seem to be the most unique and genetically isolated populations within Orissa. The two Austro-Asiatic tribes Juang and Saora were not significantly different from each other and both showed least number of alleles even at the most polymorphic STR loci such as, D21S11, D18S51, Penta E and FGA (Table 2a, 2b) and lowest heterozygosity values in as many as six loci as compared to the caste groups [30,31]. The tribal groups show relatively high between group differentiation that probably can be attributed to reproductive isolation and drift. This finding is consistent with similar studies carried on tribal populations of Central India [6]. The low heterozygosity estimate of tribes suggests that they have probably undergone some stochastic processes that have resulted from limitations in mating practices and socio-cultural differences in them.

The significantly low coefficient of differentiation among the seven populations (Fst: 0.010, p < 0.05), along with the number of alleles shared between them, confirms admixture and suggests an increased genetic affinity among populations residing in geographic proximity irrespective of their socio-cultural affiliation [3,38,39]. This is also substantiated with the AMOVA and Structure results, which showed that all the individuals of the studied populations cluster in one group and could not be subdivided further. The inability of STRUCTURE analysis to subdivide populations may be due to gene flow among groups or may be that more number of samples and loci are required to identify such close genetic subgroups.

Among the caste groups, Orissa Brahmin showed close affinity to the other upper caste populations of North India rather than to its geographic neighbors. The affinity between Bihar Brahmin and Orissa Brahmin was supported with moderate bootstrap values in the phylogenetic tree (Figure 4), which could be attributed to gene flow between them because of sharing same hierarchical status in the Hindu caste system [9]. This observation corroborates prevalent historical accounts, which suggests that the Brahmin populations of different parts of the subcontinent were natives of upper Gangetic region, who later dispersed to different parts of the country to propagate their cultural and religious ideologies and to explore better economic opportunities [15]. The phylogenetic tree (Figure 4) also clearly depicted that Khandayat and Gope are genetically more related to each other than to other occupationally similar populations (Rajput, Thakur, Maratha and Yadav) of adjoining regions. These results are in congruence with the observations of Majumder *et al *1998, where populations studied from widely separated geographic areas were found to exhibit closer genomic affinities with their geographic neighbors than with those sharing similar social ranks. It also substantiates the suggested origin of Khandayat from skilled individuals drawn from peasantry and aboriginals of the region [14]. Because the natives were assimilated into the caste system, they adopted the language and culture of the expanding and dominant upper caste population as a consequence of 'elite-dominance'. Their gene pool, however, still remains closer to aboriginals of the region. Therefore, except Brahmins, other groups were probably pooled from the local people to serve the needs of upper castes in the brahminical society. Thus, two castes bearing similar names simply represent affiliation to the same profession, but have probably different genetic constitution in different geographical regions. When populations of diverse geographic regions were included, the genetic difference among populations of the Indian subcontinent increased. This can probably be ascribed to drift caused by limitations imposed on social mobility between groups due to differences in culture and language. Juang and Saora speak Austro-Asiatic languages while Paroja follow the Dravidian language, both of which are unrelated to Oriya and by itself is a branch of Indo-European linguistic family spoken largely by the caste groups. PC analysis (Figure 3) revealed distinct isolation of the tribes from the Oriya speaking caste populations. The position of Juang and Saora in the NJ tree suggests that they are genetically still separate from other populations and extent of admixture in them from neighboring caste groups is negligible. It is also discernible that genetic distance among tribes is more strongly correlated with their genetic origin, with Paroja forming a close cluster with Madia Gond, a Dravidian tribe of India. This also substantiates the historical account describing Paroja to be an offshoot of the Gonds, one of the largest tribal populations of India. The NJ tree clearly shows that ethnic affiliation (caste/ tribe) and genetic ancestry are the key factors in shaping the genetic variation and sub-structuring among populations in geographic contiguity.

## Conclusions

Our study on linguistically distinct but geographically contiguous populations of Orissa using autosomal microsatellite markers reveals a significant amount genetic homogeneity in them. AMOVA results suggest that linguistic differences probably play a negligible role in the present day scenario in restricting gene flow between these populations. The middle-order caste groups shared genetic affinity with the local people of the area, while the Brahmins were similar to those from northern regions. Tribal populations, on the other hand, because of their long-term isolation and mating patterns, were well differentiated from the upper caste groups. This paper provides evidence that for populations living in geographic contiguity, ancestry is the governing factor in fine-tuning of genetic differentiation.

## Methods

### Population samples analyzed

Blood samples were collected from randomly chosen consenting volunteers, distributed across 17° -48' and 22° -34' North latitude and 81° -24' and 87° -29' East longitude of Orissa (Figure 1). A total of 404 individuals from seven populations, Brahmins (n = 57), Khandayat (n = 62), Karan (n = 62), Gope (n = 60), Juang (n = 50), Saora (n = 35), and Paroja (n = 78) were analyzed for the fifteen autosomal microsatellite loci. These populations were categorized based on ethnic and linguistic criteria (Table 1). The populations used for comparison in the study were selected on the basis of ethnicity, language and occupational similarity: Kanyakubj Brahmins (95), Bihar Brahmins (59), Kayastha (53), Yadav (44), Bhumihar (65), Rajput (58), Thakur (48), Irular (54), Maratha (65), Madia Gond (45), Katkari (72) and Pawara (51).

### DNA typing

DNA was extracted from blood samples using standard phenol-chloroform procedure and amplified for fifteen autosomal microsatellite loci using primers multiplexed in the Powerplex 16 System (Promega Corp., Madison, Wisconsin). STR loci analyzed in the study included thirteen tetranucleotides D3S1358, THO1, D21S11, D18S51, D5S818, D13S317, D7S820, D16S539, CSF1PO, vWA, D8S1179, TPOX, FGA and two pentanuleotides, PentaD and PentaE.

### Analysis of data

The genetic structure of the populations was analyzed at two hierarchical levels – within populations and among populations. The intrapopulation variability was estimated by analyzing the number of alleles and most common allele at individual loci and by estimating the average gene diversity [40] across the fifteen microsatellite loci. To understand the genetic variation among populations; F_ST _estimates, genetic distance and the analysis of molecular variance [41] were calculated. Genetic relationships among populations were analyzed using the Principal Component Analysis [42]. Genetic distances were estimated by using the D_A _distance measure [43], and were used to construct neighbor-joining tree [44]. The degree of support for the branches was evaluated by bootstrap analysis. To test the correspondence of genetic clusters with linguistically labeled groups, we used STRUCTURE program [45] assuming that each individual had ancestry in all clusters, so that fractions of ancestry in various clusters could be estimated.

## Authors' contributions

SS carried out laboratory experiments, statistical analysis and drafted the manuscript and VKK conceptualized the paper, provided important intellectual inputs in intrepretation of data and preparation of the manuscript. Both authors read and approved the final manuscript.
